# Fermented Cereal-based Products: Nutritional Aspects, Possible Impact on Gut Microbiota and Health Implications

**DOI:** 10.3390/foods9060734

**Published:** 2020-06-03

**Authors:** Panagiota Tsafrakidou, Alexandra-Maria Michaelidou, Costas G. Biliaderis

**Affiliations:** 1 Dairy Research Institute, General Directorate of Agricultural Research, Hellenic Agricultural Organization DEMETER, Katsikas, 45221 Ioannina, Greece; panag.tsafrak@gmail.com; 2 Department of Food Science and Technology, School of Agriculture, Aristotle University of Thessaloniki, 54124 Thessaloniki, Greece; amichail@agro.auth.gr

**Keywords:** cereal-products, fermentation, nutritional aspects, health impact, probiotics, prebiotics

## Abstract

Fermentation, as a process to increase the security of food supply, represents an integral part of food culture development worldwide. Nowadays, in the evolving functional food era where new sophisticated technological tools are leading to significant transformations in the field of nutritional sciences and science-driven approaches for new product design, fermentation technology is brought to the forefront again since it provides a solid foundation for the development of safe food products with unique nutritional and functional attributes. Therefore, the objective of the present review is to summarize the most recent advances in the field of fermentation processes related to cereal-based products. More specifically, this paper addresses issues that are relevant to nutritional and health aspects, including their interrelation with intestinal (gut) microbiome diversity and function, although clinical trials and/or in vitro studies testing for cereal-based fermented products are still scarce.

## 1. Introduction

The etymology of the term fermentation comes from the Latin verb “fevere”, which means “to boil”. Fermentation is one of the oldest known food processing methods and its history stretches back to the Neolithic period, as indicated by archaeological findings of clay tools for cheese making. Its unique ability to enhance the sensory properties of raw materials and preserve the developed product has been recognized throughout human history as miscellaneous fermented products are part of the culinary and cultural heritage of many countries globally [1,2]. This biotechnological method includes several subcategories based on primary metabolites produced: (a) alcoholic fermentation, conducted by yeasts, with ethanol and CO_2_ as the primary products; (b) acetic fermentation, conducted by bacteria of the genera *Acetobacter* with acetic acid as the primary product; (c) lactic fermentation, where lactic acid bacteria (LAB) are the fermenting microorganisms and lactic acid is the main metabolic product; and (d) ammonia or alkali fermentation of proteinaceous substrates by different *Bacillus* and *Fungi species*, with ammonia being released and giving the food a strong ammoniacal smell [3]. Their common aspect, from a biochemical point of view, is that microorganisms use their metabolic pathways to derive energy from organic compounds in the absence of exogenous oxidizing agents. Under this scope, any raw material containing organic compounds could be fermented by the microorganisms which possess the required enzymatic systems for degradation of the respective carbon sources. Recently, fermentation technology has been brought to the forefront again since it provides a solid background for the development of safe products with unique nutritional and functional attributes.

Among the available fermented products, the cereal-based ones regained popularity in line with the ever-increasing health consciousness of consumers. Cereals are essential sources of carbohydrates, proteins, minerals, fibers, and vitamins. Table 1 presents the composition of indicative whole-grain flours of various cereals. Despite their drawbacks as raw materials (i.e., deficiency in certain amino acids, lower protein content, presence of antinutrient compounds) compared to products of animal origin or dairy foods, the fermented types of cereals are nutritionally superior to their native counterparts [4].

As plant-based matrices, fermented cereal products are suitable for people with lactose intolerance, milk allergies, or people who follow a low lipid or vegan dietary pattern. They are also considered as novel probiotic delivery vehicles and potential functional foods [6]. Fermented foods’ consumption has been linked with beneficial health attributes [7] and as a whole, they represent 20–40% of the international food supply [8]. Fermentation enhances the palatability of the grains through textural and flavor changes, thus reducing the need for flavorings or other additives. Cereal grains in their raw form are characterized by low levels of organoleptically-active compounds, which corresponds to a rather flat flavor, which is often unpleasant to consumers. However, the enzymatic activity of LAB on the cereals’ components generates volatile (carboxylic acids, alcohols, aldehydes, ketones, and esters) and/or non-volatile compounds (sugars and carboxylic acids) that contribute to the sweet and sour taste and specific aroma of each product, especially in cereal-based beverages [9]. In order to promote the traditional fermented cereal products of each region in new or global markets, cross-border consumers’ acceptance needs to be met. Studies regarding consumers’ acceptance have been conducted for some products [10,11,12]. For example, Akissoé et al. [13] conducted research regarding consumers’ preference on five different types of Akpan (a cereal-based beverage from Benin). Consumers were male and female individuals, both African and European, who scored the Akpan samples using a nine-point hedonic box scale regarding the taste, appearance, and overall acceptance. Recorded differences between the Africans and Europeans highlight the importance of these types of consumer studies to re-engineer traditional products and succeed in new markets penetration.

**Figure 1:**
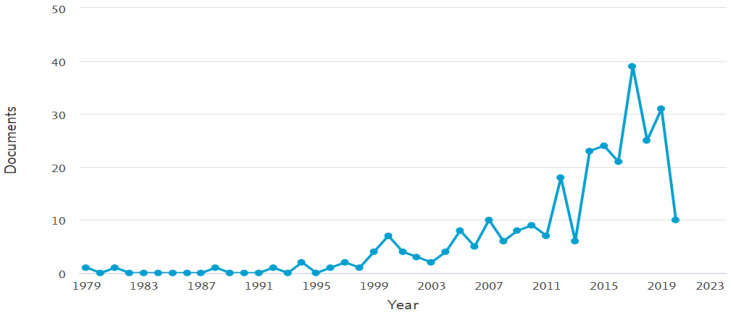
Number of articles published on fermented cereal-based products over the period 1979–2020, according to the Scopus database (last accessed 23/04/2020, document search: “fermented AND cereal AND based AND products”).

Figure 1 depicts the growing interest in this thematic area during the last decade. This could be attributed to the significant advancements in various branches of science, like food science, medicine, biology-biotechnology, genetics, etc. and to the technological innovations linked with the development of analytical methods and tools to enlighten compositional and functional elements previously identified as “black boxes”.

This paper aspires to present the latest findings of research that is being conducted regarding the nutritional aspects and functional properties of fermented cereal-based products, emphasizing the traditionally prepared foods and excluding alcoholic beverages which follow a discretely differentiated production technology.

## 2. Nutritional Aspects of Cereal-Based Fermented Foods

### 2.1. Impact on Food Safety and Shelf-Life Extension

Fermenting microorganisms employed to generate new products with improved sensorial and nutritional qualities often produces various metabolites that inhibit the growth of spoilage and/or pathogenic bacteria. These metabolites include organic acids such as lactic acid, propionic acid, acetic acid, etc. that decrease the initial pH value, creating an acidic environment in the food matrix and therefore extending the shelf-life of the fermented product [7]. Furthermore, ethanol and hydrogen peroxide, which are strong inhibitory factors for microbial growth, as well as other secondary metabolites that can act as antimicrobial compounds, are produced by some LAB and yeast species. These metabolites can be effective in controlling fungal growth and mycotoxins production in grain matrices; the latter is of great importance for cereal derived products, raising public health concern since exposure to mycotoxins may cause adverse health effects to humans [14].

*Lactobacillus* and *Pediococcus* strains, possessing antimicrobial activities, were tested regarding their efficiency to reduce mycotoxin production from *Fusarium* as well as to restrain the growth of other mycotoxigenic fungi during malting of wheat grains (used for beverages and bakery products). LAB reduced the fusarium toxins (deoxynivalenol-vomitoxin-, T-2, HT-2, and zearalenone) by up to 75%, depending on the strain. Antifungal activity was also observed from LAB metabolites (especially from acetic acid and secondarily from lactic acid) [15]. Naturally fermented sorghum, used to produce whole grain Ting (an African traditional food), was compared to similar products fermented with the addition of *Lactobacillus fermentum* strains as starter cultures. The content of mycotoxins (found in the whole grain of sorghum raw material) was decreased in all fermented samples. More specifically, *L. fermentum* FUA 3321 reduced the studied mycotoxins by up to 98% [16]. Moreover, traditional fermentation processes reduced the mycotoxin content of kunu-zaki and pito (two popular traditional cereal-based African beverages) by 59% and 99%, respectively [17]. The antifungal properties of various *Lactobacillus* strains have been tested in-situ to evaluate their effectiveness to improve the quality and safety of fermented cereal products. It is believed that the antifungal effect of LAB is the result of synergistic interactions among numerous metabolites, including fatty acids, peptides, and organic acids [18].

Antimicrobial peptides, bacteriocins, are produced by LAB and are partially related to the extended shelf-life of fermented products [19]. M’hir et al. [20] reviewed the enterococci strains that were isolated from fermented cereal products and their potential usage as starter cultures according to their technological, functional, and safety characteristics. The antimicrobial activity of 63 LAB isolates from a spontaneously fermented beverage (ogi) was explored. *Pediococcus* sp. strain OF101 showed the highest antibacterial activity against several tested food pathogens (*Bacillus cereus, Staphylococcus aureus*, *Listeria monocytogenes*, *Enterococcus faecium*, and *Escherichia coli*) [21]. Likewise, Chinese fermented foods, including sweet fermented rice, homemade sourdough, and koji, were the samples from which 132 LAB isolates were obtained. All isolates exhibited antimicrobial activity and reduced the five indicator pathogens (*E. coli* ATCC 25922, *Salmonella enteritidis* ATCC13076, *Salmonella typhimurium* ATCC14028, *L. monocytogenes* EGD-e, and *S. aureus* ATCC29213) by 2–4 log CFU/mL [22]. Consumption of traditional fermented cereal products in Africa resulted in reduced diarrhea outbreaks in children by 40% and improvement in well-being [7]. These findings indicate that isolated microbial strains from indigenous products may be used as starter cultures for standardized production of the respective cereal-based fermented product after their evaluation for technological and probiotic properties [18] or as sources to isolate metabolites and use them as pure antimicrobial agents [6].

### 2.2. Enhancement in Nutritive Value and Compositional Changes of Fermented Cereal Products

In the following subsections, an overview of the nutritional value and compositional changes due to the fermentation of cereals is presented.

#### 2.2.1. Protein and Carbohydrate Digestibility

Protein digestibility depends on the protein structure and the presence of antinutrient factors (protease inhibitors, phytases) that bind with them as well as other parameters such as pH, temperature, and ionic strength, all of which are directly related to proteolytic activities. Fermentation may affect these factors and parameters and thereby contribute to a more effective digestibility of plant proteins [23]. Proteins need to be broken down to amino acids or even small peptides to enter the human bloodstream after their absorption by the enterocytes of the small intestine, otherwise they reach the large intestine where they are fermented by the gut microorganisms, giving rise to the formation of amines and short-chain fatty acids. These fermentation products elicit various biological reactions via different receptors and mechanisms, including signal transduction involving biogenic amines as neurotransmitters and modulation of inflammatory responses [24].

Upon fermentation, microbial proteases are released and degrade to a certain extent the proteins included in a composite food matrix like cereal grains. Furthermore, the improvement of protein digestibility is accomplished through the reduction of antinutrient factors [25]. The role of phytate and polyphenols and how fermentation affects them is further analyzed in Section 2.5 (Reduction of antinutrients and allergens). Subsequently, the inactivation of digestive enzymes’ inhibitors (trypsin and chymotrypsin inhibitors) in fermented cereal products is being discussed.

The effect of fermentation on trypsin and chymotrypsin inhibitors was evaluated in bread samples made from various cereals (wheat, whole wheat, rye mix, and mixed flours). In the case of rye mix bread, the final trypsin inhibitors were almost half compared to the concentrations measured in the respective raw flour. An interesting observation was that whole wheat flour dough and bread did not exhibit any trypsin inhibitory activity, which was attributed to the bran content and the formation of complexes between the bran’s polysaccharides and the proteases inhibitors [26]. Montemurro et al. [27], studied the effect of germination and fermentation on the nutritional value and functional and technological properties of sourdough. Overall, bread made of sprouted and fermented flours showed high protein digestibility and low starch availability. Similarly, the in vitro starch and protein digestibility of fermented sorghum flour by LAB was improved. The LAB strains used have previously been isolated from fermented maize and sorghum and were chosen based on specific characteristics, e.g., pH tolerance, acidifying activity, and salt tolerance [28]. Natural fermentation enhanced protein and starch digestibility of complementary products (foods other than breast milk or infant formula—in the form of liquids, semisolids, and solids—introduced to an infant to provide nutrients) prepared from different ratios of flour blends from sorghum, millet, pumpkin, and amaranth seeds [29].

Carbohydrates digestibility is of great importance as well since it is related to many human health issues. Starch digestion is directly linked to the glycemic index (GI) of foods and concerns diabetic and health-conscious consumers who prefer products that do not rapidly raise glucose levels in blood and therefore avoid risks of insulin resistance and type 2 diabetes. Sourdough fermentation alters the cereal matrix through the production of lactic acid, which aids interactions between starch and gluten. These interactions result in reduced starch availability and a lower GI for the product [25]. Autochthonous LAB were used to ferment quinoa flour sourdough. The latter replaced the semolina needed to produce pasta by 20%. The predicted GI was lower with the fermented product, compared to the control pasta (made of wheat flour) [30]. It is worth mentioning that amylolytic LAB are scarce and limited species possess the gene amyA, which is responsible for the production of the extracellular enzyme α-amylase; actually, the formation of lactic acid from starch depends on the expression of this gene. The most efficient lactobacilli (e.g., *Lactobacillus amylovorus*) regarding starch fermentation ability belong to the genera *Lactobacillus, Lactococcus,* and *Streptococcus* [31].

Patients suffering from irritable bowel syndrome (IBS) are instructed to avoid cereals and follow a diet with low content in easily fermentative sugars. These sugars include “Fermentable Oligo-, Di- and Mono-saccharides and Polyols”, FODMAP in short. Fructans, galactans, lactose, fructose, sorbitol, and mannitol are not absorbed in the small intestine and are rapidly fermented by gut bacteria when they reach the large intestine, thus inducing abdominal symptoms (diarrhea or constipation, swollen belly, meteorism, abdominal pain, etc.) [25,32]. During fermentation, LAB, yeasts, and fungi totally or partially degrade FODMAPs. Regardless of the extent of degradation, the decrease of FODMAPs is beneficial for people with IBS symptoms, since their adverse effects depend on FODMAPs’ intake dose [33]. There are various published data regarding the content of cereal products in such sugars [34,35,36,37], but only a few reported studies are concerning the production of cereal-based products with low-FODMAP content. Struyf et al. [38] reduced the FODMAP content in whole wheat bread by more than 90% by the addition of *Kluyveromyces marxianus* and baker’s yeast (*Saccharomyces cerevisiae*) in the dough. Control bread made only with *K. marxianus* did not reach a desirable loaf volume due to insufficient CO_2_ production. Therefore, the co-culture of the two yeasts is essential for products with acceptable sensory characteristics (volume, texture, flavor) and low FODMAPs content. Menezes et al. [39] emphasized the need for combining microbial enzymatic activities from LAB and yeasts to produce added value bread that suits special dietary needs and do not lack in quality attributes. To this end, single sourdough yeast isolates were tested on their FODMAP reduction activity and CO_2_ production in a model system simulating wheat bread fermentation. *S. cerevisiae* and *Torulaspora delbrueckii*, obtained from Austrian traditional sourdough, were the most effective yeast species regarding the degree of degradation of the fructans and having the highest CO_2_ production capacity [40]. The research findings from these studies point to the potential of employing mixed cultures (LAB and yeasts) to produce palatable bakery products with improved nutritional and leavening characteristics.

#### 2.2.2. Dietary Fiber Modification

According to the American Association of Cereal Chemists (AACC), dietary fibers (DFs) are plant carbohydrates that are resistant to hydrolysis by human enzymes but can be fermented by microorganisms in the large intestine. They are classified in soluble and insoluble DFs and their ratio in food products plays a significant role in both health implications and physical–technological properties [41].

Cereals contain various DFs, which chemically and compositionally differ depending on the type of cereal and grain tissue in which they are found. The main components of DFs of cereals are non-starch polysaccharides, i.e., arabinoxylans, β-glucans, cellulose, resistant starch, fructans, and lignin, which are a phenolic polymer and often exist in composite structures with other small molecular weight bioactives, e.g., simple phenolics, minerals [42]. During fermentation of cereals, the pH is decreased due to the production of organic acids (mainly lactic and acetic) and this may result in the activation of various enzymes, either endogenous of the grains or bacterial. The enzymatic activity is responsible for biopolymer degradation, leading to grain softening (cell wall degradation) and improvement of the sensory and physiological characteristics of the fermented product [43].

Recently, the effects of fermentation *by Lactobacillus plantarum* dy-1 on barley β-glucan’s physiological and structural properties were reported [38]. Fermentation altered the state of β-glucan from a compact form (rod-shaped) in the raw barley to a smooth sheet-like structure in the fermented barley. This morphological reshaping may contribute to enhanced water adsorption or molecular binding ability. An in-depth structural analysis (NMR, FTIR, methylation analysis, monosaccharide composition) revealed that fermentation decreased the molecular weight of β-glucans (from 1.13 × 10^5^ D to 6.35 × 10^4^ D) and modified the β-(1→3) residues to the β-(1→4) residues ratio, from 1:1.98–1:2.50 in the raw barley to 1:1.8–1:2.24 in the fermented samples. These structural modifications had an impact on the physiological activities of these polysaccharides as assessed by in vitro protocols. The hypoglycemic activity was evaluated by measuring the inhibitory effect on α-amylase and α-glycosidase, inhibition of lipase was measured to determine the potential reduction of fat absorption during digestion by barley consumption, and finally, the capacity of cholesterol (re)absorption was determined for both control and fermented samples. Although all physiological activities of barley β-glucans were enhanced upon fermentation, such findings remain to be proven as actual physiological responses by in vivo testing [44]. Wheat bran fermentation by *Lactobacillus rhamnosus* 1473 tripled its water-extractable arabinoxylans (WEAX) due to the activity of endoxylanases on high molecular weight arabinoxylans [45]. The content of WEAX in flour is of great importance for the rheological characteristics of the dough and the quality of the final product [46]. Moreover, it is well known that WEAX in bakery and other cereal products increase the viscosity of the intestinal contents (digesta) and this, in turn, enhances the hypocholesterolemic and hypoglycemic potential of these food items. Zhao et al. [47] studied the impact of different fermentation procedures of wheat bran. The investigated matrix was fermented with baker’s yeast, LAB starters (*Lactobacillus bulgaricus* and *Streptococcus thermophilus*), a combination of the previous cultures, and without the addition of microorganisms (i.e., spontaneous fermentation). The effect of heat treatment (autoclave sterilization) was also evaluated. The content of WEAX significantly increased in all fermented samples by three to four times, compared to the untreated wheat bran. Soluble dietary fiber (SDF) content was also increased in fermented wheat brans, which is in accordance with data regarding SDF in sourdough production from rye [48]. A combination of fermentation and enzymatic pretreatment can thus valorize cereal brans to produce modified high fiber flours, with technological properties similar to those of refined flour streams. Overall, certain bakery products with improved nutritional value can be produced, ameliorating the undesired effects of untreated bran addition (hardness, low loaf volume, negative sensory features) [49].

#### 2.2.3. Vitamins

Vitamins play a crucial role in proper metabolic functions and therefore, their daily intake is essential since they cannot be synthesized at adequate amounts (or at all) in the human body. These essential micronutrients are divided into two sub-categories according to their solubility: a) the water-soluble vitamin C and the group of B vitamins (thiamin-B1, riboflavin-B2, niacin-B3, pantothenic acid-B5, pyridoxine-B6, biotin, folic acid, cobalamin-B12), and b) the fat-soluble vitamins A, D, E, and K [50]. Although cereals contain specific vitamins, fermentation with LAB or yeast strains can increase their vitamin content [7,8]. The ability of LAB to produce vitamins is strain-specific and these microorganisms could be used as starter or added cultures to fortify naturally fermented products for targeted nutritional and quality improvement. Such a fortification is of great importance for specific population groups that follow special types of dietary regimes, either by choice (e.g., vegans) or due to cultural habits, religious beliefs, and lack of other available food sources (developing countries). It is worth mentioning that in some African countries, porridges made of cereals can complement breastfeeding [51,52].

Biofortification in vitamins of fermented cereal products has been attempted by a few researchers [53]. For example, the incorporation of *Lactococcus lactis* N8 and *Saccharomyces boulardii* SAA655 in idli batter (an Indian steamed cake made from rice and legumes) increased the riboflavin and folate content by 40–90% [54]. Sourdough bread and pasta (with a pre-fermentation step) that were fermented by two *L. plantarum* strains showed a threefold and twofold increment, respectively, in their vitamin B2 content. The used strains were isolated from durum wheat flour and have been characterized as riboflavin-overproducing microorganisms according to in vitro tests with synthetic media [55]. More recently, Bationo et al. [52] reported that processing steps like debranning, soaking, and wet-milling may cause a decrease in cereals’ folate content by up to 60%, while on the contrary, fermentation increased folate’s concentration by up to 27%. Various fermented cereal-based foods were studied (fritters, dumplings, porridges, and gelatinized doughs, made from sorghum, corn, or pearl millet) and their estimated folate bioaccessibility ranged from 23% to 81% using an in vitro digestion model. According to Chaves-Lopez et al. [31], spontaneous maize fermentation may enhance the concentrations of nutritional compounds (thiamine, folate, riboflavin, total carotenoids, vitamin C, and vitamin E), but further preparation steps of the traditional foods result in significant decrements of each important nutrient.

Raw cereals and cereal-based fermented products are a valuable source for the isolation of vitamin producing strains. Carrizo et al. [56] isolated, identified, and evaluated the B-group vitamin production of LAB from quinoa grains and quinoa sourdough. The microbial isolates presented phytase activity, riboflavin, and folate production. Consequently, these strains could be used as ideal starter cultures to produce fermented quinoa (or other cereal) foods with enhanced nutritional value. *Propionibacterium freudenreichii* produced active B12 vitamin to an adequate level when malted barley flour, barley flour, and wheat aleurone were used as fermentation substrates [57]. In situ production of B12 by *P. freudenreichii* was also achieved with the fermentation of wheat bran [58]; co-fermentation using both *P. freudenreichii* and *Lactobacillus brevis* was investigated in this study to assess the microbial composition at the end of the process. Although co-fermentation resulted in lower concentrations of vitamins (also dependent on pH control throughout the fermentation process), the presence of *L. brevis* was essential to inhibit the growth of *Enterobacteriaceae* and *B. cereus* and thus, to ensure the microbial safety of the bran dough [58]. Greppi et al. [59] studied 151 isolates from four species (*L. fermentum, L. plantarum, Pediococcus acidilactici*, and *Pediococcus pentosaceus*) obtained from a millet-based fermented food. Their results indicated that besides the strain’s characteristics, folate production depends significantly on the duration of fermentation and the composition of the medium.

Furthermore, there are limited reports on the effect of bacterial and yeast/fungal fermentation of cereals on vitamin E concentration. Stale rice was fermented with *Cordyceps sinensis* and vitamin E increased 100%, compared to the control unfermented sample [60]. Lisosan G^®^, a nutritional supplement made from wheat germ and fermented bran with a mixed culture of LAB and yeast strains, in feeding trials using rabbits resulted in increased vitamin A and E levels in blood and showed reductions of triglycerides, LDL cholesterol, and blood reactive oxygen metabolites (antioxidant activity) [61].

#### 2.2.4. Phenolic Components

Phenolic components, which are secondary plant metabolites, are also found in notable amounts in cereals [60]. The metabolic pathways (shikimate, phenylpropanoid) for their biosynthesis involve many biomolecules like acetyl CoA, malonyl CoA, pyruvate, acetate, and some amino acids (phenylalanine and tyrosine) [16]. Their beneficial properties in human health (anti-diabetic, anti-cancer, anti-inflammatory, anti-microbial, anti-oxidant, as well as neuro-, cardio-, and hepato-protective function) are attributed to their ideal chemical structure, which promotes electron transfer or hydrogen donation from the hydroxyl groups of their aromatic ring and thereby exhibit free radical scavenging activities and metal-chelating potential [60,62,63]. Phenolic components need to be in a soluble form to enter the human blood circulation system and bring about their antioxidant properties. Phenolics in cereals can be found as free and soluble, conjugated and soluble (bound with sugars and sterols), and non-soluble, which are usually linked to polymers like arabinoxylans and lignin [63,64]. Increases of cereals’ phenolic content can be achieved by size reduction of the particles, germination, addition of hydrolytic enzymes, and fermentation [63]. Fermentation is reported to also enhance the antioxidant activity of the phenolic fraction [65]. The conditions during fermentation (temperature, final pH value, duration), microorganisms involved, as well as the type of cereal and the grain tissue employed play an important role in the outcome concerning the release of the bound phenolics [62,66,67].

Fermentation of millet with a mixture of *Lactobacilli*, mainly *Lactobacillus sanfranciscensis*, *Lactobacillus pentosus*, and yeast strains in a ratio of approximately 100:1 increased the total phenolic content by 30%, thus improving the nutritional and functional value of this underutilized cereal [68]. Wheat bran and oat bran were used as solid-state fermentation substrates by yeast (*S. cerevisiae*). Total phenolic content (TPC), composition of extracted phenolics, and antioxidant activity (in vitro) were evaluated. The best results were obtained at three days of fermentation, with a high correlation between antioxidant activities and the highest TPCs [62]. Quinoa sourdough, fermented with autochthonous lactic acid bacteria (previously isolated from the same pseudo-cereal), was used to nutritionally enrich white wheat bread. Total phenols and antioxidant activity were significantly higher in the samples containing the fermented quinoa preparation [69]. The effect of fermentation on the TPC and antioxidant activity of maize [70], oat [71], and rye [72] has been highlighted in numerous studies [25,73]. Oats are a rich source of polyphenols and avenanthramides in particular. The latter phenolic alkaloids have been reported to exhibit anti-inflammatory, antioxidant, antiatherogenic activities, and other health benefits [74]. After 4 days of fermentation, solid-state yeast fermentation of oat bran increased the apparent avenanthramide content by 48.5% and the ferulic acid content by 21.2%, which is most likely due to degradation of the compact grain cell wall matrices. The same process also increased the total phenolic content of wheat bran, where the highest percentages were obtained on day 3 [75]. Adebo and Medina-Meza [76] have published a comprehensive review of the impacts of fermentation on antioxidant activity and specifically the fate of phenolic compounds of whole cereal grains. They conclude that fermentation could positively alter not only the nutritional value of whole-grain foods, but also their sensory characteristics to allure consumers. A thorough investigation of the bioavailability of phenolic compounds concerning different fermentation media and conditions used, as well as the type of culture employed (microbial strains), is still needed to support the health benefits originating from the phenolics fraction in fermented products made from cereals.

### 2.3. Cereal-Based Fermented Foods as Probiotic Carriers

Nowadays, the market value of functional foods and beverages is growing with an average rate of 8.6% globally [77], leading the related industries to a quest of alternative and novel products to meet the consumers’ needs, stay competitive, and ensure their future survival in the globalized functional foods arena. Probiotics have been associated with dairy products for many years, but milk presents many drawbacks as a raw material according to the new trends of the food sector. From an environmental point of view, production of dairy products is generally considered among the possesses that have the greatest burdens based on life cycle assessment analysis [78]. In addition, lactose intolerance and allergies related to milk proteins concern the vast majority of the world’s population and is an issue that producers should take seriously when developing functional dairy products. Other shortcomings of dairy product matrices include the high cholesterol and fat content, cultural and strict religious beliefs of specific populations that prohibit the consumption of such foods, new diet trends, like veganism, in developed countries, as well as limited access or storage capability of dairy products in developing countries [77,79,80]. Other products that may also serve as probiotic carriers are meat, fish, chocolate, vegetables, fruit, and cereals. For the purposes of the present review, cereal-based products will be further discussed in this context.

As previously stated, cereals, containing a plethora of nutrients and bioactive compounds, are perceived positively by consumers as a healthy food choice and are cultivated and consumed all over the world. This is indicated by the overabundance of traditional fermented cereal-based foods as summarized in Table 2. However, the production of such food items, including drinks, is often characterized by uncontrolled conditions, which enhances the growth of mixed microbial populations. The predominant microorganisms in these products belong to LAB, but limited information concerning their probiotic features was available until now. Unstandardized production conditions and variances of the type and quality of raw materials complicates the procedure of identifying and studying the potential probiotic strains associated with specific end-products [80]. In vitro tests for probiotic characteristics were performed for amylolytic LAB strains, isolated from Chinese fermented cereal foods (sweet fermented rice, homemade sourdough, and koji). Three of the isolates were identified as *L. plantarum* strains and demonstrated higher or equal bile salt and acid tolerance, resistance to antibiotics, antibacterial activity, and adhesion-aggregation activities with the control commercial strain, *L. rhamnosus* ATCC 53103 [22]. Trahana (tarhana), a staple food made of wheat flour and yogurt, mainly produced and consumed in Greece, Cyprus, and Turkey, is considered to be one of the first probiotic foods [8]. Boza is another fermented beverage made of various cereals (millet, wheat, maize, rice, barley, oat, rye), which is consumed in the Balkan Peninsula, Turkey, N. Africa, and S. Russia. A varied microbial community responsible for boza’s fermentation is the consequence of the wide range of raw materials used as fermentation substrates as well as the different geographic regions (varying technologies) in which it is produced. Different bacterial species’ strains with probiotic properties have been isolated from boza [8]. Recently, a traditionally fermented sourdough from Arasbaran in Iran was used as a source for bacteria and yeasts that present probiotic properties and could be used as starter culture medium for the production of probiotic whole wheat bread. The isolates also improved the antioxidant capacity via fermentation, produced phenolic compounds, and degraded phytate, which is an important nutritional issue for whole-grain fermented products [81].

#### Health Implications of Bacteria Obtained from Cereal-Based Fermented Foods

Many of the microbial species’ strains found in fermented foods have been recognized to possess probiotic or health-promoting qualities. As a result, products containing adequate amounts of live cells of such strains may exert similar health benefits [3]. However, in vivo studies involving clinical trials are mandatory to confirm such assumptions; nevertheless, it should be highlighted that in some countries (e.g., Canada, Italy), lists of potential probiotic strains are included in regulatory guidelines, whereas in other countries (e.g., India), fermented foods with probiotic cultures are incorporated in dietary guidelines [3,88].

Besides the fortification of fermented products with nutrients and other bioactives that were previously described (vitamins, phenolic compounds), fermenting microorganisms produce additional metabolites that may exert positive effects on various health-related conditions. The expression of the genes responsible for these metabolites depends on the microbial species, strain, and the matrix in which the microorganism is grown (the type of product). For example, a polysaccharide with anti-tumor activity was isolated after the in vitro fermentation of barley (sequential treatment of steamed ground barley grains with amylase hydrolysis, followed by yeast fermentation using *S. cerevisiae*, and then bacterial fermentation with *Weissella cibaria*). The polysaccharide was tested in vivo and showed antimetastatic properties through the promotion of NK cells’ cytolytic activity and the activation of macrophages [89]. A *L. brevis* strain was also used to produce sourdough bread with anti-hypertensive properties. The strain was previously evaluated for its efficient production of γ-aminobutyric acid (GABA) and angiotensin-converting enzyme I (ACE). Both metabolites are related to the regulation of blood pressure [90].

### 2.4. Prebiotic Potential of Cereal-Based Fermented Foods

The most recent and refined definition of prebiotics was published by The International Scientific Association for Probiotics and Prebiotics (ISAPP) in an expert consensus document. The new definition expands the meaning of prebiotics from non-digestible oligosaccharides of food origin to “a substrate that is selectively utilized by host micro-organisms conferring a health benefit” [91]. In addition, the site of the host organism, where the beneficial bacteria may be located, is not limited to the human lower gastrointestinal tract (GIT), but also includes other targets such as skin, the whole GIT including the upperparts and mouth, as well as the urogenital tract [92]. Consequently, besides carbohydrates, other compounds could be considered to exert prebiotic effects, such as micronutrients (inorganic compounds), peptides, phenolics, and fatty acids [93].

Up to date, the investigated sources of prebiotic compounds are of plant origin from western countries, especially whole grains, fruit, and vegetables, i.e., wheat, barley, Jerusalem artichoke, leek, asparagus, garlic, onion, chicory root, and bananas. However, it is considered essential to explore a broader variety of raw materials and traditional products of eastern regions as potential novel prebiotic “pools” [91]. Fermented cereal-based products could be examined as promising sources of compounds with prebiotic effect since they may contain soluble fibers (β-glucans and arabinoxylans and their oligomeric products), galacto-oligosaccharides (GOSs), fructo-oligosaccharides (FOSs), resistant starch, phenolics, peptides, etc., depending on the cereal that was employed as a substrate for their production and the extent of fermentation they have undergone [93,94]. Although there are a lack of reports regarding the prebiotic effect of fermented cereal products, recent studies demonstrate a substantial effect of cereals on human gut microbiota. Tamura et al. [95] studied the utilization mechanisms of mixed-linkage β-(1,3)/β-(1,4)-glucans (MLGs) by human gut *Bacteroidetes* (*Bacteroides ovatus*). Their findings emphasize the great importance of these commonly occurring polysaccharides in cereal grains for gut microbial metabolism. Structural differences (molecular characteristics) of arabinoxylans (AXs) derived from various cereals (corn, barley, wheat, rye, rice, and oat) and the impact of different extraction methods on molecular features of these polysaccharides have been discussed by Wang et al. [96]. It is worth mentioning that the gut microbial composition may change with an alteration on the arabinosyl substitutional position in the xylan backbone, implying a possible relation between the fine structure of the polymeric carbohydrate and the composition of the microbial community. An interesting opinion paper, published lately, summarizes the intervention studies on humans which report the effects of AXs and arabinoxylo-oligosaccharides (AXOS) on various metabolic parameters. Bread and bakery products (muffins, rolls) were used as the delivery matrix of AXs and AXOs in most cases [97]. Furthermore, the beneficial role of polyphenols, which were bound to fibers of whole-grain wheat, on the inflammation of people with suboptimal dietary and lifestyle behaviors has been confirmed. The replacement of refined wheat with whole-grain wheat for an 8-week period led to a significant increment of dihydroferulic acid (DHFA) and fecal ferulic acid (FA) and a bacterial community modification [98]. Both in vitro and in vivo studies showed the inhibitory effect of dextrins from maize starch on *Firmicutes,* a bacterial phylum which is related to obesity; i.e., the *Firmicutes/Bacteroidetes* (F/B) proportion is increased in obese people compared to lean people and tends to decrease with weight loss. In parallel, the growth of microbes belonging to *Actinobacteria* and *Bacteroidetes* was stimulated, indicating the beneficial role of dextrins in healthy gut microbiota. Clinical studies are mandatory to support all these assumptions [99,100].

Besides the prebiotic fibers of the cereals, microorganisms used for fermentation may excrete such compounds. Currently, research studies focus on specific LAB (*Leuconostoc, Lactobacillus, Streptococcus, Weissella*) that produce long-chain extracellular sugar polymers (EPS). There is extensive research on the technological and functional attributes of these polysaccharides in bakery products [101]. For example, a recent study showed that EPS obtained from *L. plantarum* has a positive effect on the stalling and retrogradation of bread made from wheat sourdough when added at a 1.5% level [102]. Concerning the possible health-related benefits, only a few data are available. A wild-type strain of *W. cibaria* was used to produce a dextran with a high polymerization degree and the latter was assessed for its prebiotic potential compared to inulin. Batch fermentations using this dextran as a sole carbon source and fecal microorganisms as inoculum revealed the enhancement of *Prevotella* and *Bacteroides* populations and the increment of propionate production. Contrary to inulin, high DP dextran does not seem to stimulate bifidobacteria [103]. Wolter et al. [104] investigated the effect of flour type in sourdoughs on EPS production by *W. cibaria* MG1. Buckwheat, oat, quinoa, teff, as well as wheat flour were used as substrates to prepare sourdough fermented by the aforementioned strain. Low concentrations of fermentable sugars in oat did not favor the growth of the microorganism. The best results regarding the sourdough performance and EPS yields were obtained with buckwheat and quinoa flour, underlining the positive effects of *W. cibaria* on the production of high-quality gluten-free bread. The effect of fermentation substrate on EPS production was also investigated by Kajala et al. [105]. Two strains of *Weissella confuse* were used to ferment rye and wheat bran. The yield of the produced dextran was higher in the case of rye bran, which was attributed to the longer acidification lag phase compared to the wheat bran fermentation by both strains. An interesting approach to the preservation of the prebiotic content in gluten-free bread was recently published. Inulin is often incorporated in various foods to enhance their dietary fiber content, but also to exert other technological functionalities. Yet, the endogenous or added inulin content is reduced during the breadmaking process due to the activity of yeasts’ invertase, which hydrolyzes fructans. In this context, Morreale et al. [106] investigated the effect of inulin’s degree of polymerization as well as the use of yeasts with low invertase activity on the final inulin content and the technological characteristics of gluten-free bread made from rice flour. Bread made with the conventional baker’s yeast presented an inulin loss of up to 40% (depending on the DP of the added inulin) after baking, whereas the respective losses reached only 5% for the bread made with the low invertase activity yeast.

Polyphenols also play an important role in the modulation of gut microbiota since only a low percentage (up to 10%) of their daily intake is bioavailable, while the rest is led to the large intestine where the microbial population of the gut enzymically degrades these compounds [107]. Low-molecular-weight bioactive components are the result of such enzymic breakdown, which in turn alters the biodiversity of the gut microbiota and exerts health benefits for the host. It should be underlined though that the possible prebiotic and/or health-promoting effects of polyphenols are closely dependent on the individuals’ gut microbiome [108]. Regarding the prebiotic effect of cereals’ phenolic components, intervention studies with whole grain wheat and maize showed that bifidogenic bacteria and/or LAB were preferentially favored with limited or no changes in the numbers of the total gut microbial community. As whole-grain cereals are also rich sources of dietary fibers, it is controversial whether these effects were solely due to the phenolic compounds alone [109]. In an in vitro fermentation simulating the digestion from mouth to small intestine, Kristek et al. [110] studied the impact of 1% and 3% intake of oat bran, β-glucan extract, and polyphenol extracts separately. The results showed that the polyphenol mix increased the *Enterobacteriaceae* family within 24 h, but the most significant impact (increment of bifidobacteria, enhanced production of propionic and acetic acid) on the gut microbiota was achieved with the digestion of oats and not by a single group of compounds; this implies a synergistic effect of the dietary fibers and polyphenols on gut microbiota and also points to the lack of energy for microbial growth in the case of using single compounds as substrates.

### 2.5. Reduction of Antinutrients and Allergens

Besides valuable nutrient compounds, cereals contain a notable number of components that are considered anti-nutritional factors (ANFs). These components include phytic acid/phytate (myoinositol-1,2,3,4,5,6-hexakis dihydrogen phosphate), tannins, and polyphenols [25]. Phytate or phytic acid is a secondary metabolite, found mainly in the aleurone layer and pericarp (wheat, rice) or in the endosperm (maize) of cereals, serving as their phosphorus repository. The content of phytate in cereals varies between 0.18 g to almost 6.5 g per 100 g product (dw) [111]. The presence of phytic acid plays a crucial role in the nutritional value of the food in which it is found as it has the ability to hinder enzymatic activity (trypsin and beta-galactosidase) and form chelates with metal ions, i.e., iron, magnesium, calcium, and zinc, thus reducing their bioavailability [112]. Likewise, tannins and polyphenols have a strong negative effect on protein digestibility since their hydroxyl groups form complexes with the carbonyl group of proteins. As a result, proteins precipitate, proteases are inhibited, and thereby, amino acid deprivation is realized when a diet is based on cereal products rich on polyphenols, which is the case in most developing countries or for people following a vegan dietary pattern [7,23]. Prolonged periods of these nutritional deficiencies may also lead to osteoporosis, iron deficiency anemia, and impairments of physical growth [113].

Among the technologies and strategies applied for ANFs reduction, fermentation is considered one of the most effective ones. Mineral binders were reduced by fermentation in togwa [114], sorghum porridges [115], and finger millet porridges [51]. Phytic acid can be degraded by endogenous or microbial phytases during natural fermentation and increase the bioavailable amount of iron, calcium, and zinc. The value of pH encountered for fermented products is considered optimal for the enzymes to act against phytate [111,112,116]. Wet and solid-state fermentation of various cereals and their brans have been studied considering the physicochemical alteration of the products and the effect on dephytinization. Spaggiari et al. [45] investigated the use of *L. rhamnosus* 1473 strain in a solid-state lactic acid fermentation of wheat bran to increase its nutritional profile. Indeed, phytic acid decreased to 36.4% due to the fermentation process. Similar findings were also reported by Zhao et al. [47], where over 20% of the phytic acid contained in the wheat bran was degraded using yeast and LAB solid-state fermentation. In both research approaches, high temperatures were applied as pretreatment methods and therefore endogenous phytases are believed to be deactivated. The authors concluded that dephytinization could be attributed exclusively to microbial phytases. Phytic acid degradation has also been studied in other cereal-based fermented products. *Lactobacillus sanfrancisco* CCM 7699, *L. plantarum* CCM 7039, *L. amylovorus* CCM 4380, and *L. plantarum* CCM 7039 were used for fermentation of wheat flour in two tarhana samples, boza, oat, and rice beverages, respectively. Over 80% of phytic acid was degraded in both tarhana samples after 144 h of fermentation (6 days is the mean time needed for tarhana preparation), while complete degradation was observed after 10 h of fermentation in all fermented beverages [117]. Recently, the effect of fermentation’s temperature on phytic acid degradation and the mineral content in whole-wheat sourdough bread was reported. A combination of starter cultures (*Lactobacillus* sp.) at the lowest tested temperature (25 °C) gave the best results concerning nutritional value and sensory properties [118]. Furthermore, pseudo-cereals have also been reported to benefit from fermentation regarding the bioavailability of their minerals. Quinoa, canihua, and amaranth flours following fermentation exhibited improved accessibility of iron, calcium, and zinc compared to their raw counterparts [119].

The synergistic effect of seed germination and fermentation on nutritional and functional aspects has been explored by Montemurro et al. [27]. Among other raw materials, wheat, barley, and quinoa were subjected to germination, drying, and milling. The flours were then used to prepare sourdough bread. Flours obtained after seed sprouting were characterized by lower phytate and tannin content, whereas the produced bread showed higher protein digestibility compared to conventional wheat flour bread. The tannin content was reduced by 30–39% in grain sorghum using fermentation, steaming, and flaking processing, indicating the amelioration of the biological value of sorghum protein compared to control samples [120].

An equally important issue is the reduction of cereal allergenicity through the fermentation process. Food allergies are a globally major concern since they seem to affect almost 5% of adults and 8% of children under 18 years [121]. In general, all food proteins may cause allergic reactions; however, gluten proteins from wheat and related grains (barley, rye, and possibly oat) are considered to be among the most common sources that trigger allergies [122]. More specifically, gliadins (α-, β-, γ-, ω-), low molecular glutenins (LMW), high molecular glutenins (HMW), and the albumin/globulin fractions are involved in the mechanism (IgE-dependent) that induces cereal allergenicity [123]. It should be emphasized that celiac disease (CD), also known as celiac sprue and gluten-sensitive enteropathy, differs from a cereal proteins allergy and is rather a food intolerance, encountered in almost 1 out of 100–300 Caucasian individuals worldwide. Celiac disease (an autoimmune disorder) is characterized as an inflammatory disease of the upper small intestine in genetically predisposed persons triggered by the consumption of products that contain gluten [123,124]. Hydrolysis of proteins by microbial or endogenous proteases and peptidases, occurring during fermentation, may result in diminished allergic reactions through the degradation of the respective antigenic epitopes [19]. Although there are many reports regarding the technological properties and nutritional enhancement of cereal-based products after fermentation, current research concerning the effects of this ancient process on their allergens is rather limited. Di Cagno et al. [125] prepared a sourdough bread, which was made from wheat and a mixture of non-toxic flours from oat, millet, and buckwheat. As starter microorganisms, selected strains of lactic acid bacteria (*Lactobacillus alimentarius* 15M, *L. brevis* 14G, *L. sanfranciscensis* 7A, and *Lactobacillus hilgardii* 51B) were used based on their ability to hydrolyze gliadin fractions. The same research group performed a pilot study with young celiac disease patients. They showed that bakery products made from wheat flour and the aforementioned microbial strains are non-toxic to CD patients [126].

An in vivo study with CD patients was performed and results showed that the consumption of bread with the selected bacteria and nontoxic flours did not affect intestinal permeability. Moreover, an in vitro study examined the effect of sourdough lactic acid bacteria on IgE-binding proteins in bread made from wheat and rye flours. Compared to control samples (bread made with baker’s yeast), the tested products showed that the IgE-reactive epitopes were significantly decreased [127]. The capacity of VSL#3 (a mixture of probiotic strains) to ferment wheat flour and reduce its toxicity was investigated. The findings indicated that gliadins were degraded to a large extent, meaning that these microorganisms could be used as effective cultures for sourdough preparation [128]. It appears that during fermentation of sourdough by *Lactobacillus sp*., gluten’s disulphide bonds are reduced, which further enhances the enzymatic activity of cereal proteases and hence, gluten digestibility is improved [129,130].

## 3. Effect of Cereal-Based Fermented Foods’ Components on Gut Microbiota

Gut microbiota contributes to a wide spectrum of human health aspects. These numerous and variable microbial species (bacteria, viruses, archaea, eukaryotes) may reach 10^14^ cells in the gut and their composition can be affected by genetic factors, lifestyle, diet, stress, diseases, and the use of pharmaceutical products [131,132,133]. A beneficial, stable, and well-balanced composition is crucial for the maintenance of immune equilibrium, the integrity of the gut epithelial cells, and inflammation prevention. Dysbiosis of the gut ecosystem is associated with intestine related diseases like IBS, skin inflammation (psoriasis, atopic dermatitis), cardiovascular diseases, cancer, obesity, mental health, arthritis, and type II diabetes [2].

Currently, the available intervention studies regarding fermented cereal-based products focus on the impacts of sourdough bread on specific GI diseases as bread is one of the most consumed products around the globe [134]. Therefore, there are no published data yet on the ways that cereal-based fermented foods may act on human gut microbiota and the possible modifications that they may cause on the species’ dynamics. On account of this, scientific evidence of specific food components and their impact on human gut microbial consortium will be shortly discussed herein.

Fermented products in general and cereal-based foods in particular are characterized by an abundance of ingredients that reach the GI tract and are accessible by the gut microorganisms of the host. These ingredients include macronutrients such as carbohydrates and proteins, micronutrients like vitamins, phenolic compounds, and minerals, but also bacterial components from the fermentation cultures, biogenic metabolites (organic acids, biogenic amines, etc.), or even live microorganisms (probiotics) that interact with the human microflora located in the gut [135].

The daily intake of fermented food and beverages ranges between 5% and 40% depending on the geographic area. Viable bacteria from fermented foods along with foodborne microbes from other food sources may reach up to 1.0% of the commensal gut microbiota (10^10^ to 10^11^ ingested bacteria per day). These transient microorganisms are eliminated through feces, but can also adhere to the GI tract and eventually alter the gut microbial composition [2,131]. Fermented foods enrich the diverse intestinal microbiome, mainly with gram-positive bacteria belonging to the *Firmicutes* and *Actinobacteria* phyla, such as *Lactobacilli, Lactococci, Streptococci, Enterococci*, *Carnobacteria, Bifidobacteria, Brevibacteria*, and *Propionibacteria*, and some yeast and fungi species (*Saccharomyces, Kluyveromyces, Debaryomyces, Penicilium*, etc). Subsequently, dietary habits may exhibit a strong effect on intestinal bacterial dynamics through the consumption of various viable microorganisms. The latter could have an inhibitory or stimulating effect on the gut autochthonous species [2,136].

Dietary choices may also alter the microbial balances in the gut, as is evident from data obtained from clinical studies testing various diets (western, plant-based, meat-based, Mediterranean, vegan, and vegetarian) [137]. Animal-based diets (low fiber content and high animal protein and fat) seem to decrease the population of *Firmicutes* and on the contrary, increase bile-tolerant species (*Alistipes, Bilophila,* and *Bacteroides*) [136]. Likewise, gluten-free diets increased the numbers of *Enterobacteriaceae,* i.e., *E.coli* and other potentially pathogenic bacteria [138]. Conversely, diets based on plant-based foods (vegan, vegetarian) or balanced diets (Mediterranean) were linked with higher counts on “beneficial” bacteria (*Lactobacillus* sps. and *Bifidobacterium* sps.) [139]. It should also be mentioned that these diets include elevated daily intakes of fibers, a better ratio of mono- and poly-unsaturated to saturated fatty acids (compared to animal food-based diets), and a variety of antioxidant compounds [137].

## 4. Concluding Remarks and Future Perspectives

Fermentation, a traditional food processing technology, is gaining attention again due to the recognition of significant benefits it delivers to the products. Furthermore, since it has low energy requirements and no complicated equipment is needed, it is considered a viable solution for developing countries to support local economies, valorize local raw materials, and provide products with valuable compounds as a solution to malnutrition. To surpass the safety issues that may occur from the lack of training, proper facilities, low quality of local resources, and problems in the logistics chain (from production to consumption), a standardized procedure for each “traditional” product must be determined and selected starter cultures should be used, following optimization strategies for all parameters affecting the outcome of the fermentation process. Microbial domestication and technological properties of the selected strains are considered crucial and therefore, should be studied thoroughly to be proposed for large or domestic scale efficient production of fermented cereal products.

**Figure 2:**
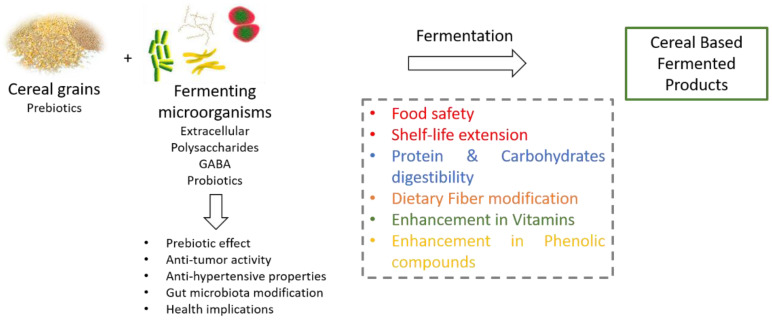
Schematic diagram of the main effects of fermentation on cereal-based products.

The present review covers the recent literature regarding the main nutritional aspects of cereal-based fermented foods (Figure 2).

The effects of fermentation on protein and carbohydrate digestibility, reduction of antinutrients and allergens, and enhancement in phenolic compounds and vitamins are discussed. In addition, the role of cereal-based fermented products as probiotic carriers as well as their prebiotic potential is presented. The aspect of each trait on human health promotion and well-being is presented in the respective section. Technological implications of fermentation on the properties of end products, such as the impact on food safety and shelf-life extension, along with modifications on the nature and content of specific compounds are reviewed.

Although there is sufficient evidence for the positive contribution of these products in human health, including alleviation of medical disorders, there is a need to unravel the underlying mechanisms. This must proceed by properly designed clinical studies to evaluate in-depth possible cause and effect relationships, involving physiological responses linked with chronic diseases. Meticulous data interpretation will be needed to establish the position of cereal-based fermented products in national dietary guidelines and provide useful information for tailored functional foods production. The latter would not only meet the growing demand of consumers in developed countries for healthier or “natural” foods, but will also lighten the burden of hunger and undernourishment in many developing countries.

## Figures and Tables

**Table 1:** Composition of whole-grain flours (adapted from [5]).

Type of Whole-Grain Flour	Energy (Kcal/100g)	Protein (%)	Total Lipids (%)	Ash (%)	Carbohydrates (%)	Total Dietary Fiber (%)
Barley	345	10.5	1.6	1.28	74.52	10.1
Buckwheat	335	12.62	3.1	2.54	70.59	10
Corn	361	6.93	3.86	1.45	76.85	7.3
Millet	382	10.75	4.25	1.21	75.12	3.5
Oat ^1^	404	14.66	9.12	1.97	65.7	6.5
Rice-brown	363	7.23	2.78	1.54	76.48	4.6
Rye-medium	349	10.88	1.52	1.2	75.43	11.8
Soft Wheat	332	9.61	1.95	1.53	74.48	13.1
Sorghum	359	8.43	3.34	1.32	76.64	6.6
Teff	366	12.2	3.66	N.A. ^2^	70.73	12.2

^1^ Partially debranned, ^2^ N.A.: Not Available.

**Table 2:** The most common traditional cereal-based fermented foods and beverages worldwide.

Name	Cereal Used	Fermentation Microorganism (s)	Country/Region of Origin	Type of Product	Reference
Boza	Millet, Wheat, Maize, Rice, Barley, Oat, Rye	LAB	Bulgaria, Albania, Turkey, Republic of Northern Macedonia, Romania, S. Russia, N. Africa	Beverage	[8]
Bushera	Sorghum, Millet	LAB	Uganda	Beverage	[8,77]
Cheka	Sorghum, Maize	Unknown	SW. Ethiopia	Beverage	[82]
Chicha	Maize	LAB, Acetobacter	Peru	Beverage	[83]
Dhokla	Rice	*Leuconostoc mesenteroides, L. fermentum,* *P. pentosaceus, Pichia silvicola S. cerevisiae*	India	Steamed Cake	[8]
Dosa	Rice	*L. mesenteroides, Streptococcus faecalis, L. fermentum, Bacillus amyloliquefaciens*	S. India	Pancake-Like	[8]
Idli	Rice	*L. mesenteroides, Lactobacillus delbrueckii, L. fermentum, L. lactis, S. faecalis, Pediococcus cerevisiae, L. plantarum* *Subsp. plantarum, L. pentosus, L. plantarum spp. argentoratensis*	India, Sri Lanka	Steamed Cake	[8]
Injera	White or Red Sorghum, Tef, Wheat, Barley, Finger Millet Or Maize	*Pullaria sp., Aspergillus sp., Penicillium sp., Rhodotorula sp., Hormodendrumsp., Candida sp., L. bulgaricus*	Ethiopia, Africa	Pancake-Like	[8,84]
Kenkey	Maize	*L. fermentum, L. reuteri*	Ghana	Sourdough Dumpling	[7,8]
Khambir	Wheat	Yeast, Mold, LAB, *Bifidobacterium sp.*	W. Himalayas	Flat Bread	[85]
Kishk	Wheat, Oats	*L. plantarum, L. brevis, Lactobacillus casei, Bacillus subtilis, Yeasts* *L. rhamnosus, Lactobacillus sakei*	Egypt, Syria, Arabic Countries	Soup	[8]
Kisra	Sorghum	*P. pentosaceus, Lactobacillus coprophilus,* *Lactobacillus cellobiosus, L. brevis, L. fermentum, L. amylovorus, Lactobacillus reuteri, Candida intermedia, Debaryomyces hansenii,* *S. cerevisiae*	Sudan	Pancake-Like	[8]
Koko	Maize	*L. plantarum, L. brevis*	Ghana	Beverage	[7]
Kvass	Rye	*L. casei, L. mesenteroides,* *S. cerevisiae*	Central Europe	Beverage	[8]
Kwunu-Zaki	Millet, Sorghum, Maize	LAB	Northern Nigeria	Beverage	[7]
Liha	Maize	Unknown	Ghana, Togo, Benin, Nigeria	Beverage	[7]
Mahewu	Millet, Sorghum, Maize	*L. delbrueckii, L. bulgaricus, Streptococcus lactis*	S. Africa, Togo	Beverage	[7,77]
Mangisi	Millet	Unknown	Zimbabwe	Beverage	[7]
Mawe	Maize	LAB	S. Africa, Togo	Dough	[7,8]
Munkoyo	Sorghum, Millet Or Maize Plus Munkoyo Roots	Unknown	Zambia, Africa	Beverage	[7]
Mutwiwa	Maize	LAB	Zimbabwe	Porridge	[7]
Ogi, Ogi-Baba	Maize, Millet, Sorghum	*L. plantarum*	Nigeria, W. Africa	Pudding	[7,8]
Pozol	Maize	*Streptococcus bovis, Streptococcus macedonicus, L. lactis, Enterococcus sulfureus, L. fermentum*	Mexico, Guatemala	Beverage	[8]
Tarhana, Trahana	Wheat (Rye, Maize, Barley, Corn, Oat, Buckwheat)	*L. bulgaricus, S. thermophilus, L. lactis,* *Lactococcus diacetylactis, L. acidophilus, Lactococcus cremoris, L. casei, S. cerevisiae*	Greece, Cyprus, Turkey	Soup	[8]
Tobwa	Maize	LAB	Zimbabwe	Beverage	[7]
Togwa	Sorghum, Millet, Maize	*Lactobacillus, Streptococcus*	Tanzania	Beverage	[7,77]
Uji	Maize, Millet, Sorghum	*L. plantarum*	Uganda, Kenya, Tanzania	Beverage	[7]
Ricera	Rice	*S. thermophilus, L. acidophilus, L. bulgaricus, Bifidobacterium bifidum*	Unknown	Unknown	[76]
Koozh	Millet, Rice	*Weissella paramesenteroides, L. plantarum, L.* *fermentum*	S. India	Beverage	[77,86]
Kunu	Maize, Millet, Sorghum	*Lactobacillus, Lactococcus, Leuconostoc, Pediococcus,* *Weissella*	W. Africa	Beverage	[87]
